# Spark Plasma Sintering and Electrospark Deposition of High Entropy Alloys with Elemental Variation

**DOI:** 10.3390/ma18122799

**Published:** 2025-06-13

**Authors:** Ciprian Alexandru Manea, Laura Elena Geambazu, Ileana Mariana Mateș, Delia Pătroi, Gabriela Beatrice Sbârcea, Dorinel Tălpeanu, Jan Přikryl, Gifty B. Oppong, Augustin Semenescu

**Affiliations:** 1 National Institute for R&D in Electrical Engineering ICPE-CA Bucharest, Splaiul Unirii 313, 030138 Bucharest, Romania; ciprian.manea@icpe-ca.ro (C.A.M.); delia.patroi@icpe-ca.ro (D.P.); gabriela.sbarcea@icpe-ca.ro (G.B.S.); dorinel.talpeanu@icpe-ca.ro (D.T.); 2 Faculty of Material Science and Engineering, National University for Science & Technology POLITEHNICA of Bucharest, 313 Splaiul Independentei, 060042 Bucharest, Romania; augustin.semenescu@upb.ro; 3 Central Military Emergency University Hospital “Dr. Carol Davila”, Calea Plevnei 134, 010825 Bucharest, Romania; 4 Gerosion ehf, Árleynir 2-8, IS-112 Reykjavik, Iceland; jan@gerosion.com (J.P.); gifty@gerosion.com (G.B.O.); 5 Academy of Romanian Scientists, 3 Ilfov St., 050044 Bucharest, Romania

**Keywords:** HEA, spark plasma sintering, electrospark deposition, coatings

## Abstract

A novel processing route of producing CrFeNiMo, Co_0.5_CrFeNiMo, and Al_0.5_CrFeNiMo high-entropy alloy coatings was proposed in this work. Pre-alloyed HEAs were consolidated by spark plasma sintering (SPS) in order to fabricate electrodes for electrospark deposition (ESD) coatings on carbon steel substrates. Investigations were performed to observe aspects such as phase composition and stability, contamination level, homogeneity, elemental distribution, and microstructural integrity. The results indicated phase stability and lower porosity when increasing the SPS temperature by 50 °C for all cases, with tetragonal distortion related to the HEAs’ severe lattice distortion core effect. The coating surface analysis indicated that a continuous and compact coating was obtained, correlated with the ESD layering count, but microfissures were present after 6 layers were applied due to the reduced ductility combined with rapid cooling under Ar atmosphere. The chemical integrity of both the sintered samples and the coatings was preserved during the processing, revealing a uniform elemental distribution with no contaminations or impurities present. The results indicated successful HEA sintering and deposition, highlighting the potential of the combined SPS-ESD process for high-performance material fabrication with possible applications in aggressive environments.

## 1. Introduction

Lately, both surface repairing [1,2] and improvement methods [3,4,5] are applied to high-value components operating in aggressive environments to enhance the physical and mechanical characteristics [6,7,8]. In the surface repair context, physical and microstructural characteristics that limit the effectiveness of traditional fusion welding are exhibited by certain materials, especially when hot-cracking could occur [9,10,11]. Alternative methods must be investigated in the attempt to avoid the replacement of an entire damaged part.

In order to prevent or delay the initiation of surface defects, various traditional surface treatment processes such as physical vapor deposition (PVD), chemical vapor deposition (CVD), electroplating, or thermal diffusion (TD) could be applied. The techniques are widely used for improving surfaces in terms of hardening and wear and corrosion resistance properties [12], but some disadvantages, such as relatively high costs of deposition equipment, pollution, or long processing times, must be taken into consideration.

In the current research, Electro Spark Deposition (ESD) was investigated as a coating technique for surface enhancement of a carbon steel grade C1018 (CS) substrate aimed at improving structural integrity and environmental durability by using high entropy alloys (HEAs) from the CrFeNi system with elemental variation as coating materials [13]. This technique involves the material transfer from the electrode (deposition material) to the substrate (workpiece) by using an electric arc generated by the discharge of the power supply capacitors. Low pulse frequency along with short pulse duration allows heat dissipation, reducing the heat-affected zone without modifying the microstructure of the substrate [3]. Another advantage of the rapid cooling rates of the ESD is the ability to produce nanostructured coatings with increased hardness and tribological characteristics [14,15,16]. Depending on the electrode and substrate characteristics, such as electrical resistance, thermal conductivity and inertia, melting temperature, density, diffusivity, and chemical reactivity [17], the deposition parameters (voltage, capacitance, and frequency) require appropriate adjustments.

A wide variety of metallic materials can be deposited by this technique, from single elements or traditional alloys [18,19,20] to more complex materials such as multi-principal element alloys or high-entropy alloys [21,22,23,24,25].

HEAs were selected as coating materials due to the possibility of producing alloys with tailored properties that could withstand harsh environments, benefiting from their specific core effects: high entropy, cocktail effect, severe lattice distortion, and sluggish diffusion [26]. Recent studies have demonstrated the HEAs’ advantages when compared to conventional alloys, where high strength [27], high corrosion resistance [28], and wear resistance [29] indicate their potential application as structural materials in various domains [30,31,32]. HEA is known to offer superior strength and toughness [33,34], while ESD provides a low-cost, precise coating method with minimal heat damage and strong bonding, according to Jihui Yan et al. [34]. Literature also highlights that the AlCrFeCoNi coating produced by ESD presents excellent wear resistance and hardness, making it ideal for wear-resistant applications without the need for post-processing [35].

In terms of HEA processing methods, various techniques have been developed over the last years, which include additive manufacturing (AM) [36], mechanical alloying (MA), spark plasma sintering (SPS) [37], and vacuum arc melting (VAM) [38].

The SPS technology brings multiple benefits, which include time and economic efficiency when compared with the classical sintering methods, due to rapid heating and reduced processing time. The technique consists of pressing and sintering powder materials simultaneously in a graphite die, which undergoes a process of rapidly uniformly heating, controlled by the action of DC pulses generated by a high-power source and controlled cooling. When compared to the classic sintering methods, the SPS process is performed in an electric field, leading to a high densification degree at lower temperatures [39]; this method was selected for its relevance to the paper’s objectives. According to literature [40], the SPS technique was classified among the most efficient procedures to consolidate HEAs due to their demanding requirements, such as high densification with no defects, phase stability, and homogenous elemental distribution, but also due to the benefits of fracture toughness, stiffness, compressive strength, and ductility [41,42,43]. Hence, SPS was chosen as the consolidation technique for this work. The downselection was based on the hardness, density, and porosity results of the sintered samples.

For this paper, a novel approach of producing CrFeNiMo, Co_0.5_CrFeNiMo, and Al_0.5_CrFeNiMo HEA coatings was considered, the materials being processed from metallic powders, sintered by the SPS technique, and deposited with the aid of the ESD method. In order to assess the integrity and compositional uniformity of the ESD coatings, the surface microstructure and chemical and elemental distribution were investigated using SEM, EDS, elemental mapping, and XRD analysis. Due to their structural characteristics and phase stability, the coatings have great potential as candidates for applications in harsh or aggressive environments.

## 2. Materials and Methods

For this paper, CrFeNiMo, Co_0.5_CrFeNiMo, and Al_0.5_CrFeNiMo high-entropy alloy metallic powders were used. The description of the process and the characteristics of the milled powders were described in a previous paper. The raw metallic powders were milled for 30 h with the aid of N-heptane as a process control agent, the results indicating a sufficient alloying degree for the studies HEAs [44]. The metallic powders were sieved, and the fraction ≤ 80 µm was selected in order to avoid potential unnecessary porosity and defects in the consolidated samples.

To consolidate the obtained HEAs for further processing into ESD electrodes, the mechanically alloyed metallic powders were sintered via Spark Plasma Sintering (SPS) technology. For this research, an HP D25 FCT Systeme GmbH, Effelder-Rauenstein, Germany, equipment was used. Three sets of consolidated trial samples (diameter 20 mm, height 6 mm) were obtained for each composition. The sintering temperature parameter was varied to achieve the optimal mechanical properties of the bulk to be machined in deposition electrodes for coating purposes. The sintering temperatures (i.e., isothermal hold) were set based on the average melting temperature of the elemental materials and with slow heating and cooling rates to avoid internal or superficial tensions. The SPS parameters used for the experimentation are presented in Table 1.

The samples were sintered in high-density graphite dies that were lined with graphite foil. Graphite is used when sintering via SPS due to its excellent properties, such as high thermal stability, electrical conductivity, and high melting point. To avoid temperature losses, graphite felt was used to cover the die during the process. After the SPS process is completed, the samples are removed from the die and lining, sandblasted with glass microspheres, polished, and cleaned with alcohol in order to remove any graphite remains as well as the carbides that eventually formed during the process. Although the sample’s surface is in direct contact with the graphite components, proper mechanical and chemical cleaning reduces the possible contamination with C.

**Figure 1:**
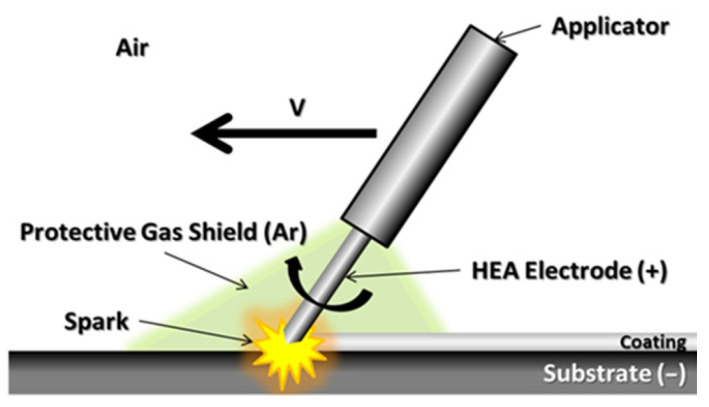
Schematic representation of the electrospark deposition process.

To produce HEA electrodes (length 40 mm, diameter approx. 5 mm due to equipment requirements), a new batch of sintered samples was produced, cut, machined to shape, and cleaned with high-purity alcohol. The deposition process was performed using the Spark Depo Model 300 (TehnoCoat, Shizuoka, Japan) equipped with a miniature applicator. The type of applicator selected for the deposition has a great impact on the roughness degree and coating quality. During the entire process, an argon atmosphere was used as a protective shield to prevent excessive oxidation. The shield is formed through a nozzle located next to the electrode and covers the deposition area and the close surroundings (see Figure 1). The deposition method consisted of applying successive metallic (HEA) layers on an electroconductive substrate, low-carbon steel grade C1018 (CS) for the present case. Low-carbon steel, grade C1018, was used as a substrate for the current case due to its present usage in the production of piping and other components for geothermal brine transfer, but also auxiliary parts in the geothermal environment.

The substrates were prepared prior to the deposition process by polishing with sandpaper (1000 grit) to remove any impurities and to achieve a uniform surface and subsequently degreased with high-purity alcohol (99.99%) after. To coat the CS substrates, tests were performed to determine the deposition parameters presented in the following Table 2. During the deposition process, the output was set to preserve the spark intensity and deposition rate.

A miniature applicator was used for the ESD process since this type of applicator aids with obtaining a fine surface with roughness between Ra = 4–12 µm according to the equipment specifications. In order to observe the layering effect of the EDS technique, 2 types of coatings were produced: 3 layers (C1) and 6 layers (C2) on the substrate surface.

In order to observe the microstructural and chemical characteristics of the bulk and coating materials, the following equipment was used: Field Emission Scanning Electron Microscope Auriga CarlZeiss SMT, 2010 (Carl Zeiss SMT GmbH, Oberkochen, Germany), extended with X-ray energy dispersive spectroscopy probe—EDS model X-MaxN (Oxford Instruments, Oxfordshire, UK); EDS Aztec analysis software (AZtec 2.1) for Scanning Electron Microscopy (SEM) (Carl Zeiss SMT GmbH, Oberkochen, Germany), Energy Dispersive Spectroscopy (EDS), and chemical mapping. X-ray diffraction analysis (XRD) was performed with D8 DISCOVER, Bruker AXS (Bruker, Rheinstetten, Germany).

The crystallographic phases were identified using the ICDD PDF 2 Release 2022 database, where the structures were similar to the following indexed files: PDF 01-081-5609—Fe_3.3_Mo_2_, PDF 03-065-5131—Fe_3_Ni_2_, PDF 01-071-8300—FeMo, PDF 01-082-3433—Fe_1.1_Mo_0.9_, PDF 00-024-0511—FeCr_2_O_4_, PDF 03-065-6712—Cr_0.99_Fe_1.01_, PDF 03-065-4528—CrFe, PDF 00-031-0404—Cr_9_Mo_21_Ni_20_, PDF 01-085-5454—NiO,PDF 03-065-4528—CrFe, PDF 00-005-0703—FeCrMo for CrFeNiMo HEA, PDF 03-065-5131—Fe_3_Ni_2_, PDF 01-071-7594—CrNi, PDF 03-065-4528—CrFe, PDF 01-071-8300—FeMo, PDF 03-065-6829—FeCo, PDF 00-056-1302—Fe_2_O_3_, PDF 00-021-0868—CoMoO_4_ for Co_0.5_CrFeNiMo HEA, PDF 01-079-8744—FeMo, PDF 00-066-0698 -Al_87.96_Cr_21.34_Ni_4.20_, and PDF 00-050-1265—AlNi_3_, PDF 01-071-9767—Mo_0.984_Ni_0.016_, and PDF 01-071-7597—Cr_0.8_Ni_0.2_ for Al_0.5_CrFeNiMo HEA.

The crystallographic phases for the HEA coatings were identified by using the ICDD PDF 2 Release 2022 database, where the structures were similar to the following indexed files: PDF 01-071-7542—(Cr_11_Fe_13_Ni_3_)Mo_3_, PDF 03-065-8992—Cr_6_Fe_18_Mo_5_, PDF 01-071-7597—Cr_0.8_Ni_0.2_, PDF 01-085-1410—Fe, PDF 01-071-9765—MoNi_4_, PDF 03-065-7277—Fe_0.875_Mo_0.125_, PDF 01-071-7568—Cr_0.5_Mo_0.5_ for HEA-FREE-C1/HEA-FREE-C2, PDF 01-071-9764—Mo_1.08_Ni_2.92_, PDF 01-071-7597—Cr_0.8_Ni_0.2_, PDF 01-077-7598—CrFe, PDF 03-065-7519—CoFe_15.7_, PDF 01-081-4998—Cr_0.8_Mo_0.4_Ni_0.8_, PDF 01-087-7825—Mo_0.11_Cr_0.26_Fe_0.63_, PDF 01-073-9565—Cr for HEA-Co-C1/HEA-Co-C2, PDF 03-065-7277—Fe_0.875_Mo_0.125_, PDF 01-077-6757—(AlFe_3_)_0.5_, PDF 03-065-8992—Cr_6_Fe_18_Mo_5_, PDF 01-077-7958—Fe_0.86_Mo_0.14_, PDF 03-065-5131—Fe_3_Ni_2_, and PDF 00-051-0637—Cr_7_Ni_3_ for HEA-Al-C1/HEA-Al-C2.

Hardness testing was performed by using FM 700 FutureTech (Kawasaki, Japan) Vickers hardness tester equipment. According to the Vickers hardness Test Standard [45] for each sample, at least 5 indentations (applied test force of 2 kgf for a dwell time of 15 s) were performed, and the average value was calculated.

To obtain the densification degree of the sintered samples, the apparent density was determined by Archimedes’ principle with an XS204 METTLER TOLEDO, (Greifensee, Switzerland) hydrostatic balance. Five sets of measurements were performed for each sample in air and immersed in 96% purity ethanol at 21 °C. Theoretical densities were calculated with Equation (1) based on the densities of the constituent materials and mass percentages. The densification degree was calculated with Equation (2):

(1) $$\rho_{t}=\frac{100}{\sum \frac{m_{e}}{\rho_{e}}}$$

where, $m_{e}$ represents element wt.% and $\rho_{e}$ represents the element density

(2) $$Densification\;Degree=\frac{\rho_{a}}{\rho_{t}}*100$$

where $\rho_{a}$ is the apparent density and $\rho_{t}$ is the theoretical density

Porosity was calculated based on the values obtained from Equation (2).

## 3. Results and Discussion

### 3.1. Spark Plasma Sintering—HEA Consolidation

The HEA metallic powder materials, CrFeNiMo, Co_0.5_CrFeNiMo, and Al_0.5_CrFeNiMo, were consolidated to produce ESD electrodes with the temperature set according to Table 1, having the end goal of obtaining the most suitable candidate for further processing.

#### 3.1.1. CrFeNiMo

**Figure 2:**
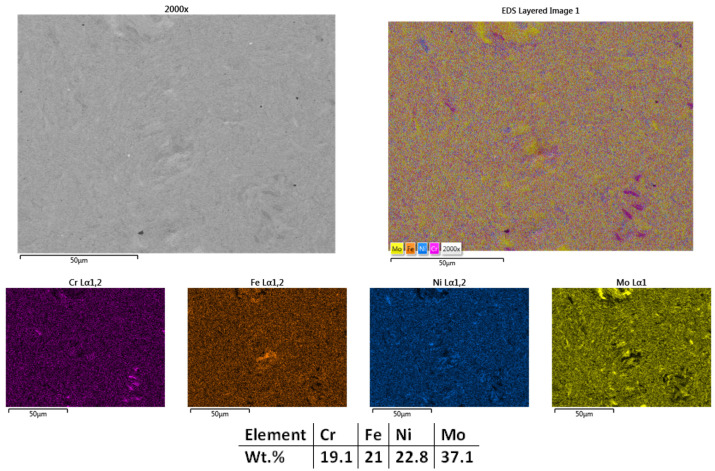
HEA-FREE-S1-950—SEM, Mapping, and EDS analyses results for CrFeNiMo HEA sintered at 950 °C.

The surface microstructural analyses results presented in Figure 2 reveal a compact structure with low porosity and no visible defects, indicating that a high degree of sintering was achieved in most regions; however, some areas exhibited minor heterogeneity in particle bonding. The elemental mapping analyses results indicate sufficient microstructural refinement, where molybdenum-rich particles are not fully bound to the alloy matrix. The EDS analyses results confirm the elemental composition, with no contamination and with the elemental concentration close to the theoretical one. Although the carbon contamination and oxidation were taken into consideration, the content is under the detection limit of the used equipment.

**Figure 3:**
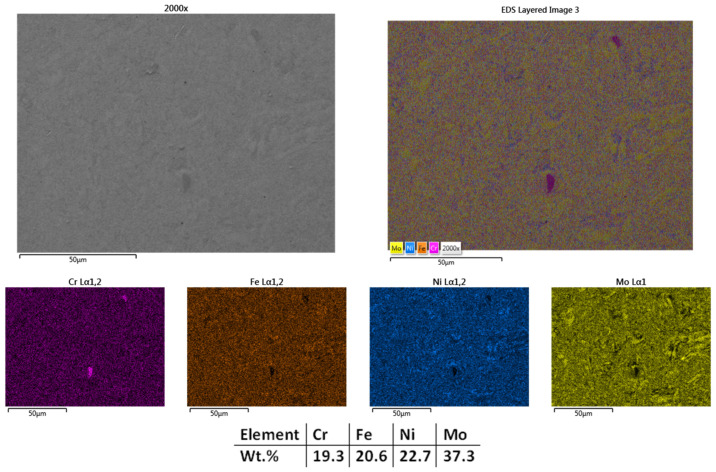
HEA-FREE-S2-1000—SEM, Mapping, and EDS Analyses Results for CrFeNiMo HEA Sintered at 1000 °C.

The next temperature used for the CrFeNiMo HEA SPS processing was 1000 °C, and the results are presented. Figure 3 presents the microstructural analyses results showing lower porosity and a higher degree of sintering than the sample sintered at 950°, indicating an improved densification of the material. Mapping and EDS analyses results present particle refinement with a homogeneous distribution of the elements across the entire analyzed area with no impurities or contaminants. The oxygen and carbon levels were under the equipment detection limit and did not influence the results obtained. Although there is an increased sintering degree, there are still isolated areas with incomplete sintering in terms of diffusion of the chemical elements within the matrix, which led to the decision to increase the sintering temperature further for this material. The SPS grain refinement attributes were reported in literature by Moravcik et al. [46] when studying the microstructure and mechanical properties of Ni_1.5_Co_1.5_CrFeTi_0.5_ HEA. It was noted that a tensile strength of 1384 MPa and bending strength of 2593 MPa were obtained, demonstrating the mechanical alloying followed by SPS consolidation efficiency when compared to casting of HEAs. In alignment with the research results, further refinement by SPS was considered for the current case.

**Figure 4:**
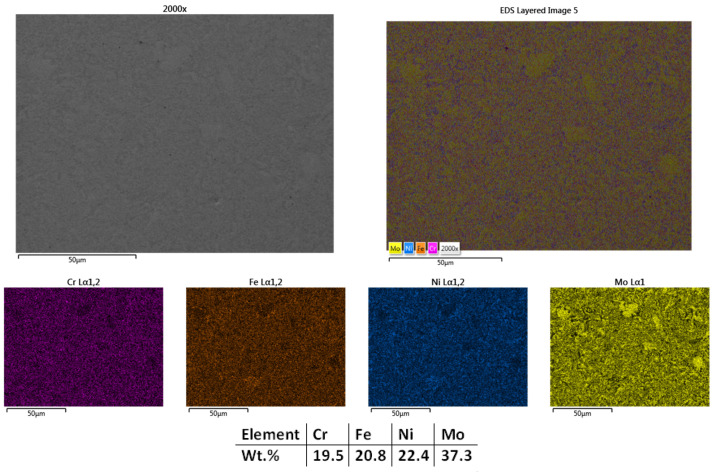
HEA-FREE-S3-1050—SEM, Mapping, and EDS Analyses Results for CrFeNiMo HEA Sintered at 1050 °C.

With the aim to further refine the microstructure, the next sample was sintered at 1050 °C, and the results showed improvement. It could be observed that the sample was sintered (Figure 4) with minimum porosity; thus, considering that the experiments regarding the sintering temperature for this composition can be concluded. Figure 4 presents the mapping and EDS analysis, which confirmed the microstructural findings, showing an overall good elemental distribution with no impurities or contamination. Similar to the previously sintered samples, there were no traces of C and O identified, but their presence was not excluded. There are no cracks present in the structure, indicating that there are no significant internal or superficial tensions caused by the process.

**Figure 5:**
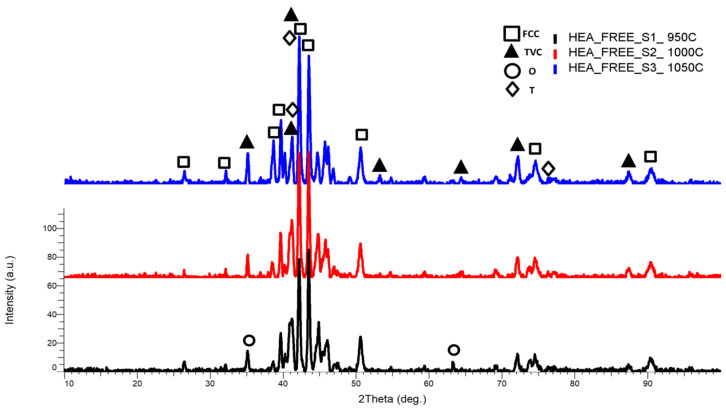
XRD comparative analysis results for the CrFeNiMo HEA sintered at 950 °C, 1000 °C, and 1050 °C.

From the XRD comparative results patterns (Figure 5), the highest intensity peak was identified at the 2θ angular position of 42.21° for the samples sintered at 950 °C and 1000 °C and at the 2θ angular position of 43.49° for the samples sintered at 1050 °C, with a major face-centered cubic phase (FCC), tetragonal phase (TVC), and trigonal phase (T) with the presence of NiO, CrNi, Fe_2_O_3_, FeMo, CrFe, and FeCrMo phases, indicating that all elemental components are present and alloyed. When comparing the results, it was observed that the peak intensity decreased with the temperature increment, indicating further alloying and lattice distortion. All major peaks are concentrated in the area of maximum intensity.

Minor phases and reflections of FCC, TVC, and T were identified at the 2θ angular positions of 26.5°, 32.1°, 40.22°, 41.2°, 49.5°, 54.71°, 72.2°, 73.69°, 87.5°, and 90.49° angles.

It is worth mentioning that in the literature [47,48], the trigonal (or rhombohedral) phase is presented as improving the properties of a material by increasing hardness, chemical stability, thermal conductivity, and more. For example, certain forms of alumina (Al_2_O_3_) are trigonal and can exhibit properties such as high hardness, making them ideal for cutting tools.

For CrFeNiMo HEA sintered at 950 °C (HEA-FREE-S1-950C), the presence of a minor orthorhombic (O) phase was identified at the 2θ angular position of 35.13° and 64.39° with an abundance of CrMoNi, this phase being a hardening phase.

#### 3.1.2. Co_0.5_CrFeNiMo

The equiatomic CoCrFeNiMo was previously studied in terms of its applicability in aggressive environments [49,50,51]. For the present case, the Co content was reduced due to the global Critical Raw Materials (CRM) situation, where the Co raw material exploitation leads to a deficit. The development of new materials can be seen as a strategy to replace (by using alternative materials that are more abundant locally and more sustainable from an ecological point of view) or reduce the use of CRMs [52].

Therefore, the second composition sintered was Co_0.5_CrFeNiMo. It is known that Co improves the high-temperature strength (heat resistance and creep resistance) and increases corrosion resistance but also wear resistance. Additionally, the mentioned benefits, Co also improves the material machinability, a property necessary for the current case where electrodes are produced by cutting and machining into shape for the ESD.

**Figure 6:**
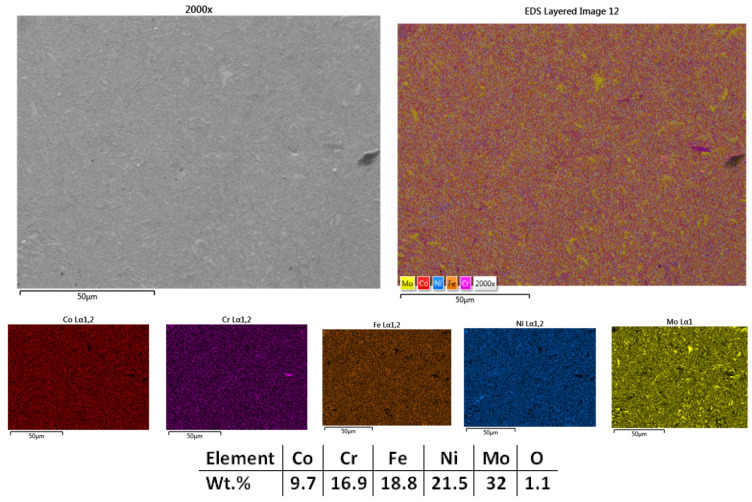
HEA-Co-S1-900—SEM, Mapping, and EDS Analyses Results for Co_0.5_CrFeNiMo HEA Sintered at 900 °C.

As presented in the microstructural analysis results from Figure 6, it can be observed that the sintering process had the expected effect on the metallic powder, the sample having a high compacting degree. There are no major defects present, with a low presence of pores, but the structure could be further refined.

The mapping analyses and EDS results (Figure 6) present a uniform elemental distribution over the analyzed area, but there are still Mo, Cr, and Ni particles with incomplete diffusion within the matrix. The EDS analysis revealed that the sample contains oxygen in the detection range of the equipment. The presence of oxygen could be justified by the in-air powder weighing and manipulation for die assembly preparation.

**Figure 7:**
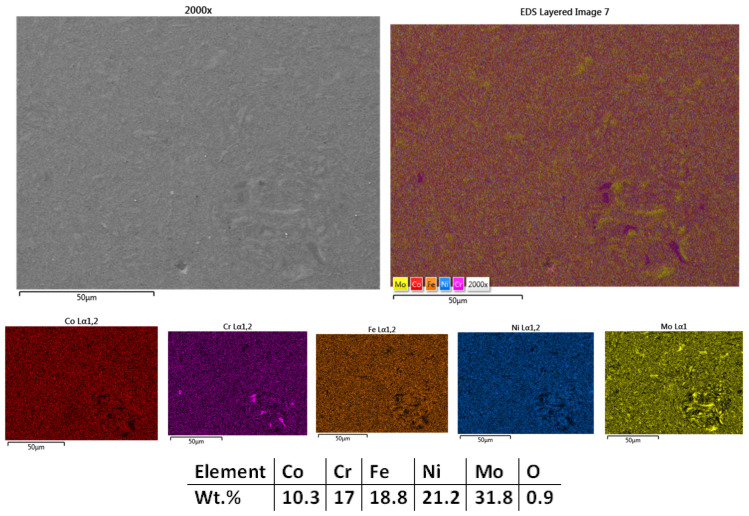
HEA-Co-S2-950—SEM, Mapping, and EDS Analyses Results for Co_0.5_CrFeNiMo HEA Sintered at 950 °C.

The microstructural results for the sample sintered at 950 °C (Figure 7) indicated that the structure was further refined and no defects were observed, while temperature increment had a notable impact on the samples. Elemental mapping results presented an improved elemental distribution over the analyzed area, with incomplete Cr and Mo sintering but with complete Ni sintering. Similar to the sample sintered at 900 °C, the presence of oxygen was noted. Based on the results, it was decided to increase the sintering temperature by 50 °C for further refining the structure.

**Figure 8:**
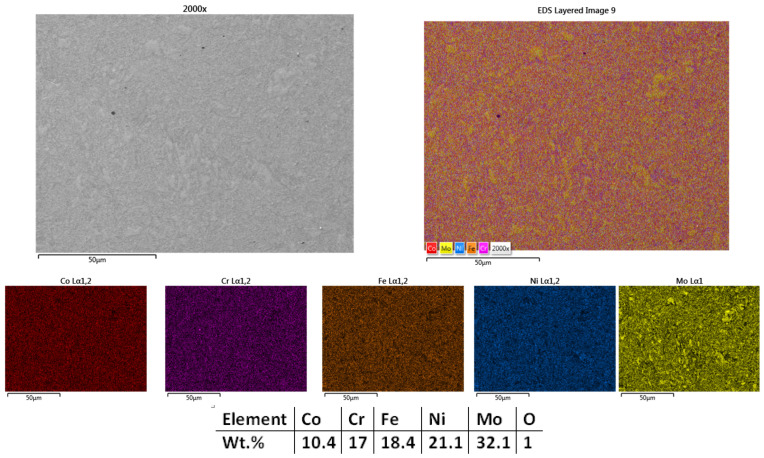
HEA-Co-S3-1000—SEM, Mapping, and EDS analyses results for Co_0.5_CrFeNiMo HEA sintered at 1000 °C.

It was observed that the most promising results were obtained for the sample HEA-Co-S3-1000 (Figure 8), which was sintered at 1000 °C. There are no visible pores and no areas with incomplete sintering. As for the other samples, no cracks were observed and no other structural defects. The elemental mapping results present an advanced sintering level for molybdenum and a complete sintering of the other elements. It was also noticed a high degree of homogenization and elemental distribution. From the EDS analysis, the composition is confirmed and close to the theoretical calculations, with no impurities and low oxidation.

Based on the microstructural and chemical analysis, the sample sintered at 1000 °C (HEA-Co-S3-1000) was identified as the most suitable candidate for further processing due to its enhanced microstructural refinement and more uniform elemental distribution.

**Figure 9:**
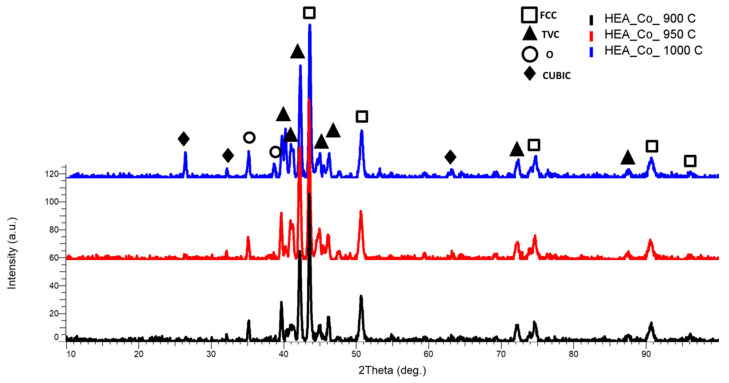
XRD comparative analysis results for the Co_0.5_CrFeNiMo HEA sintered at 900 °C, 950 °C, and 1000 °C.

The XRD comparative analysis result (Figure 9) shows that all major peaks are concentrated in the area of maximum intensity, behavior noted at the CrFeNiMo HEA as well, with the highest intensity peak identified at the 2θ angular positions of 43.5°, with an FCC major phase, with an abundance of FeNi and CrNi, and a secondary peak with a TVC phase at the 2θ angular position of 42.2°, having an abundance of CrFe and FeMo. A minor cubic phase was identified at the 2θ angular positions of 26.42°, 32.05°, and 63.08° with an abundance of FeCo and MoCo, where the MoCo combination was only identified for the sample sintered at 1000 °C. It was observed the presence of simple cubic phases at 1000 °C, being related to the temperature increment of 50 °C, indicating the continuous alloying degree improvement. The lattice distortion appears to be present in the current case too, since the BCC phase was not identified.

#### 3.1.3. Al_0.5_CrFeNiMo

The 3rd composition developed was Al_0.5_CrFeNiMo. Aluminum is known for its reduced weight due to its low density, and it is used in applications where corrosion resistance, machinability, and high strength are required. A lower content of Al could enhance properties such as toughness and heat resistance, but its presence improves ductility and provides an ideal balance of workability and hardness by reducing brittleness. The benefits of Al in the studied alloy are to be desired considering both the material application and processing techniques [53].

**Figure 10:**
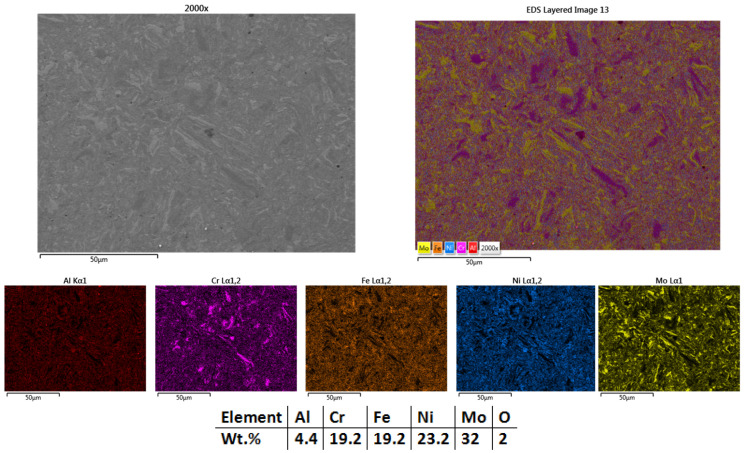
HEA- Al-S1-850—SEM, Mapping, and EDS analyses results for Al_0.5_CrFeNiMo HEA sintered at 850 °C.

Due to the lower average melting temperature, the first sintering process attempt for this composition was at 850 °C. From the preliminary microstructural analysis results, it was observed that there was incomplete sintering (Figure 10), with microfissures and pores. The temperature was deemed insufficient for the studied case, and the elemental mapping results (Figure 10) revealed incomplete sintering of the elemental materials. Overall, the sintered sample presents a good compacting degree, but the results indicated the high possibility of cracking or mechanical failure to be encountered, rendering the sample unusable in this case.

The results highlighted that a higher sintering temperature was required to obtain a higher densification degree with minimal defects. For further experimentation, it was necessary to achieve the desired electrode geometry that can withstand shearing, tearing, and breaking during the deposition process.

As for the Co_0.5_CrFeNiMo sintered HEA, oxygen was detected in the EDS analysis. Al tends to form Al oxides, which could indicate an increased corrosion resistance but also higher hardness in the final product.

**Figure 11:**
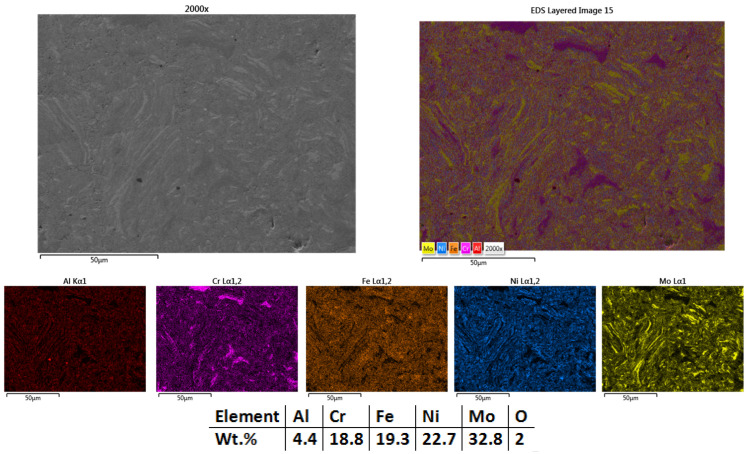
HEA- Al-S2-900 –SEM, Mapping, and EDS analyses results for Al_0.5_CrFeNiMo HEA sintered at 900 °C.

When sintered at 900 °C, a sintering improvement in terms of diffusion of the chemical elements within the matrix was observed, but it was not considered sufficient to withstand the forces that intervene during the mechanical processing into electrospark deposition electrodes or during the deposition process when the electrode is used as a functional part. The risk of electrode breaking during the deposition was taken into consideration, so it was decided to go further with a higher sintering temperature. The microstructure (Figure 11) revealed that the sample sintered at 900 °C was also not compacted sufficiently, but an improvement was observed when compared to the sample sintered at 850 °C. In Figure 11, the mapping and EDS analysis results are presented. Mo and Cr were not sintered completely, but an improvement was observed for Al and Cr sintering degrees when compared to the sample sintered at 850 °C. The temperature increment modified the microstructure and refined it, but it was not considered to be sufficient.

Good homogenization and elemental distribution were observed, and the chemical composition analyses indicated the oxygen presence was similar to the previous sample. The chemical composition of the initial mixture was confirmed, and the weight percentages are close to the theoretical calculations where no contaminants were present.

According to the results obtained after sintering the Al_0.5_CrFeNiMo HEA at 850 °C and 900 °C, the temperature plays an important role, and the microstructure could be improved at only a 50 °C increment, leading to the following results.

**Figure 12:**
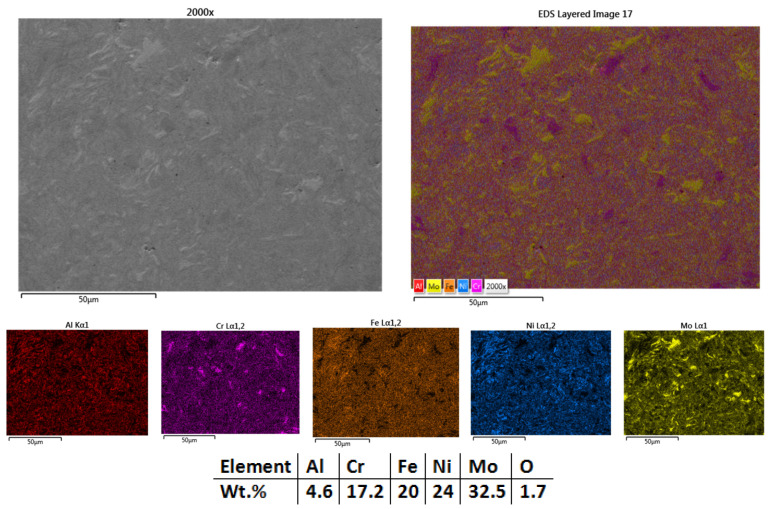
HEA- Al-S3-950—SEM, Mapping, and EDS Analyses Results for Al_0.5_CrFeNiMo HEA Sintered at 950 °C.

At the sintering temperature of 950 °C, a low pore presence was observed from analysis results along with a high degree of sintering with the absence of surface microfissures (Figure 12). This result indicated optimal sintering parameters for the present case, where the sample could be further processed into electrodes without breaking risks.

From the elemental mapping results, the improved particle refinement and sintering degree were confirmed. The EDS analysis results reveal the absence of contaminations, oxygen content in a low quantity, and validation of the desired chemical composition.

As it is known, Al has a strong affinity for oxygen, forming stable oxides such as Al_2_O_3,_ which could influence the EDS analysis results in addition to the mechanical alloying effects over the alloyed mixture. Alleg et al. [54] reported that the addition of low Al content in the Mo_0.5_NiTiZrAl_x_ (x = 0.3) HEA promoted the corrosion resistance of the alloy when tested in 3.5 wt.% NaCl solution at room temperature, due to the Al_2_O_3_ films besides the passivation of other elements such as Mo. Additionally, the corrosion resistance, the Al content improved the wear resistance due to the oxidative wear mechanism but also promoted grain refinement.

**Figure 13:**
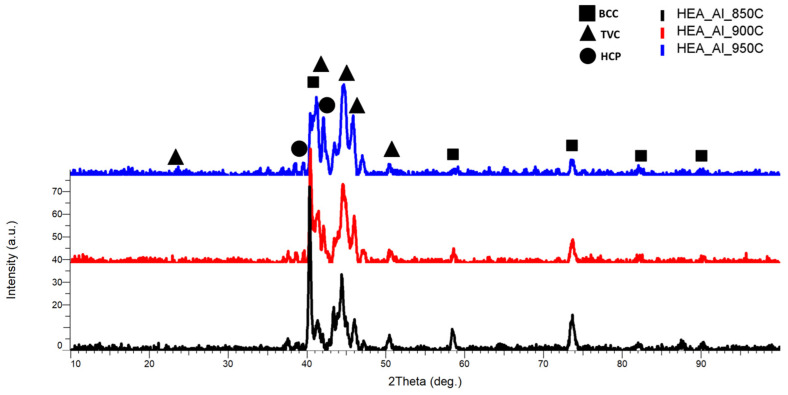
XRD comparative analysis results for the Al_0.5_CrFeNiMo HEA sintered at 850 °C, 900 °C, and 950 °C.

The XRD comparative analysis results from Figure 13 indicate an improved crystallization concomitant with the temperature increment, where for the sample sintered at 950 °C the peak identified at the 2θ angular position of 40° presents the highest intensity, with a major BCC and TVC phase with an abundance of FeMo, AlNi, MoNi, and CrNi. When comparing this alloy to CrFeNiMo HEA and Co_0.5_CrFeNiMo HEA, the presence of the HCP phase was identified at the 2θ angular position of 42°, with an abundance of AlCrNi. It was observed that an optimal ratio of strength and ductility can be induced when HCP and BCC phases coexist in the crystal lattice, which indicates that it could similarly apply in the present case based on the obtained results. Minor phases and reflections of the 3 identified phases were identified at the 2θ angular positions of 21°, 38.67°, 50.55°, 58.62°, 73.6°, 82.13°, and 90.1°. The minor phases tend to be reduced in intensity and broadened at the sintering temperature of 950 °C, whereas for the other 2 analyzed compositions (CrFeNiMo and Co_0.5_CrFeNiMo), all major peaks are concentrated in the area of maximum intensity with the presence of all elemental materials being identified.

The obtained results of the sintered HEAs are in accordance with the theory for phase stability calculations, where the valence electron concentration calculated with Equation (3) predicted a solid solution phase mixture of FCC and BCC in abundance (VEC_CrFeNiMo_ = 7.5, VEC_Co0.5CrFeNiMo_ = 7.67, and VEC_Al0.5CrFeNiMo_ = 7.0) with a ductility-strength balance according to Guo et al. [55].

(3) $$VEC=\sum_{i=1}^{n}{VEC\;}_{i}\cdot c_{i}$$

Due to the severe lattice distortion encountered as a core effect in HEA, the TVC phase could indicate a multi-phase system with FCC and BCC distortion suggesting a phase separation or partial transformation, which could be impacted by the high temperature and pressure used during the sintering process [56].

After the microstructural and chemical analysis, the samples were analyzed in terms of hardness, density, densification degree, and porosity, presented in Table 3. From the hardness testing, it was observed that the average HV tends to increase with the sintering temperature, which is to be expected for this type of process, but for CrFeNiMo HEA sintered at 1050 °C (HEA-FREE-S3-1050), a decrease was noted. The change could be due to reasons such as atomic diffusion, closed pores, or phase transformations at elevated temperatures. Although the hardness decreased, it was decided to go further with the HEA-FREE-S3-1050 due to the previous analysis results and reduced brittleness. The samples Co_0.5_CrFeNiMo HEA sintered at 1000 °C (HEA-Co-S3-1000) and Al_0.5_CrFeNiMo HEA sintered at 950 °C (HEA-Al-S3-950) had the best performances in terms of structural integrity due to the absence of defects and increased hardness.

A high degree of densification was obtained, close to the theoretical density for HEA-FREE and HEA-Co, while an increase in the density was observed for HEA-Al up to 89.014% for sintering at 950 °C. The results are correlated to the microstructural analysis results, where porosity was observed to decrease with temperature increment.

### 3.2. HEA Coatings Obtained by ElectroSpark Deposition (ESD)

Electrospark deposition, or electrospark alloying, is known as a surface engineering technology for coating production with superior adhesion to the substrate, good uniformity, and desired chemical and mechanical properties either in air or under a protective gas shield. The adhesion between the coating and the substrate takes place due to the chemical mixing of the superficial molten substrate surface with the deposition material transferred from the electrode during spark discharge, representing one of the main advantages of this technique along with the short-term thermal stress due to the applied pulsed current [57].

Past results revealed that when HEA alloys were used as coatings deposited by the ESD method, it was observed that a diffusion layer is formed between the substrate and the coating, which could withstand pull-off testing without any damaging effects [55,56]. Additionally, the good adhesion, the HEA coatings presented high corrosion resistance when exposed to a saline solution of 3.5 wt.% NaCl for 6 h [58].

**Figure 14:**
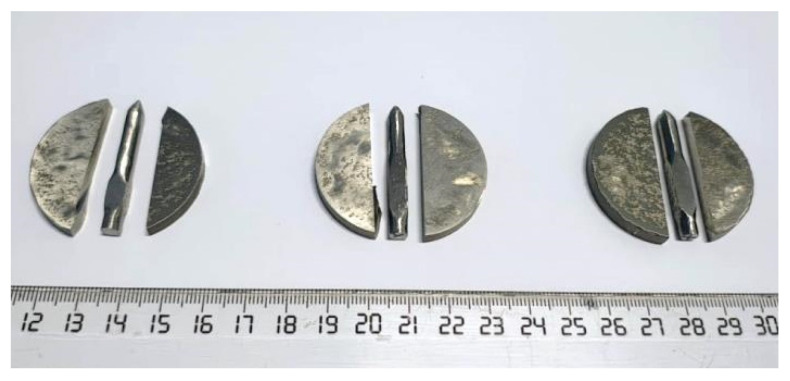
Electrodes prepared for the electrospark deposition process.

For the experimentation, the CrFeNiMo, Co_0.5_CrFeNiMo, and Al_0.5_CrFeNiMo HEAs sintered (diameter 40 mm and height 6 mm), cut, and machined into electrodes were used for the coatings production. To produce the electrodes, the median area of the samples was selected to maximize the electrode length as presented in Figure 14. The electrode’s shape was adjusted based on coating background with the electrospark deposition method [59,60].

Although a regular cylinder shape could be used, risks of fracturing, non-perfect contact, and uniformity issues were encountered. The modified electrode shape presents a hexagonal end to be fixed in the applicator holder, a broader middle area to avoid shearing, and a cone-shaped tip. The application specifications indicate maximum efficiency when the electrode forms a 30–60° angle with the substrate (according to the equipment requirements). For the current case, a 45° angle was maintained during the deposition, this implying the need for a conical-shaped tip. Lastly, the electrodes were finished with SiC paper and cleaned before the deposition occurred.

The deposition rate was calculated on a singular pass made on a 25 mm length. The deposition rate varied based on the electrode composition, resulting in deposition quality and continuity, implying a slower pass when necessary. The number of surface-deposited layers was based on the material behavior during the process, where the coated substrate was weighted after each complete layer. After a specific number of layers, a negative weight gain is observed, indicating an inefficiency in the deposition process. This method of verification was used to identify the point at which further coating became ineffective, as additional material no longer contributed to improving the final coating thickness. After this point the coating materials will not be applied efficiently, and no benefits will be brought to the final coating. For the current case, 2 variants were made with 3 (C1) and 6 layers (C2) for all 3 compositions. It was observed that after the 6th layer, the mass was constant, implying that no benefits were brought to the substrate from that point forward.

#### 3.2.1. CrFeNiMo

The first analyzed coatings were the C1 and C2 produced from the CrFeNiMo (HEA-FREE) electrode. The SEM analyses results for the coated surfaces present a uniform and continuous coverage over the surface, where superficial microcracks were observed. This type of defect is usually present on the surface, and it is specific to the electrospark deposition process due to the rapid cooling. The fissures are usually present only on the surface, and they do not cross the coating [59,60].

**Figure 15:**
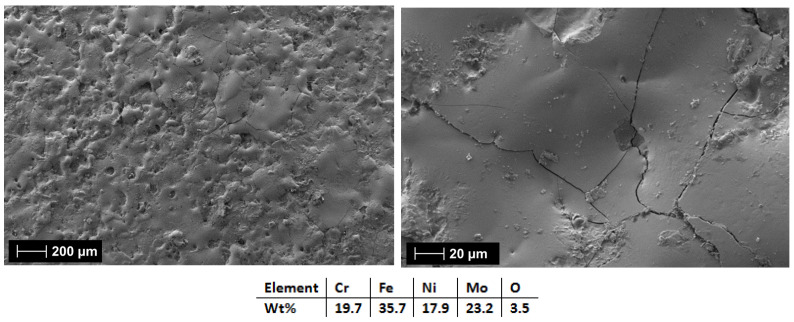
SEM and EDS analysis results for HEA-FREE-C1.

**Figure 16:**
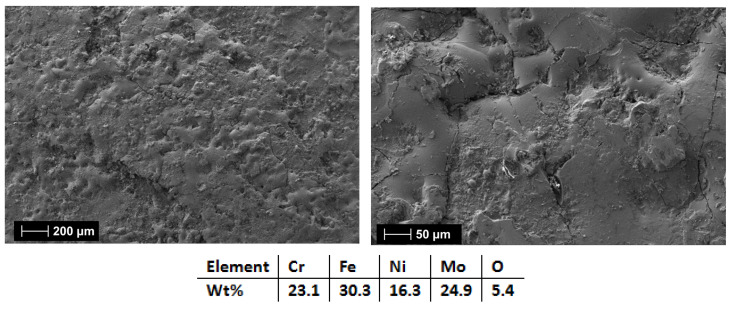
SEM and EDS analysis results for HEA-FREE-C2.

Figure 15 and Figure 16 showed that when 3 layers were applied (HEA-FREE-C1), the microfissures were not wide and the surface was not as rough when compared to the 6 applied layers (HEA-FREE-C2) coating. The roughness could have an influence on the scaling issues in aggressive environments, where a smoother surface is to be desired. The EDS analysis results confirm the coatings’ compositions with the oxygen presence. Although this method uses Ar shielding during the deposition, the process is performed in air, and the effect of slight oxidation is to be expected.

**Figure 17:**
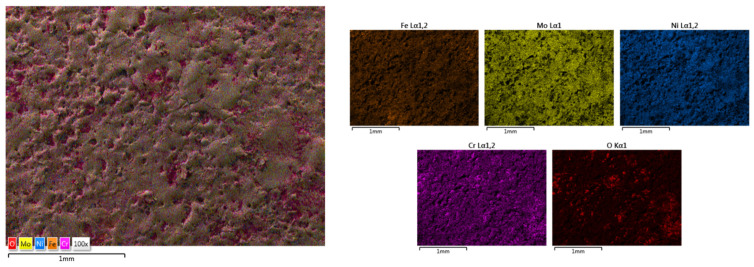
Elemental mapping results for HEA-FREE-C1.

**Figure 18:**
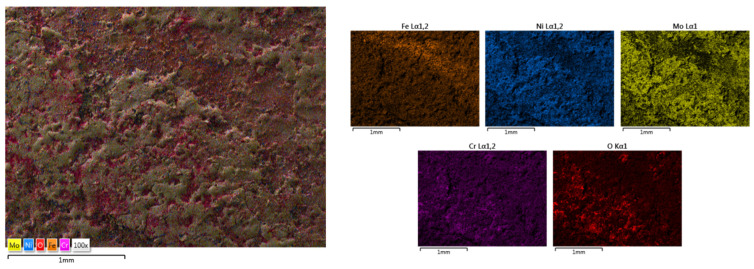
Elemental mapping results for HEA-FREE-C2.

The elemental mapping results present the elemental distribution over the surface and the overall homogeneity for both cases (Figure 17 and Figure 18). For sample HEA-FREE-C2, it was observed that areas with Fe abundance and Mo oxidation are present. Mo has the tendency of forming passive films [54], which could be beneficial when testing for corrosion resistance. The HEA-FREE-C1 sample presented a very high uniformity with lower overall oxidation, indicating better results when compared to the HEA-FREE-C2 sample.

#### 3.2.2. Co_0.5_CrFeNiMo

**Figure 19:**
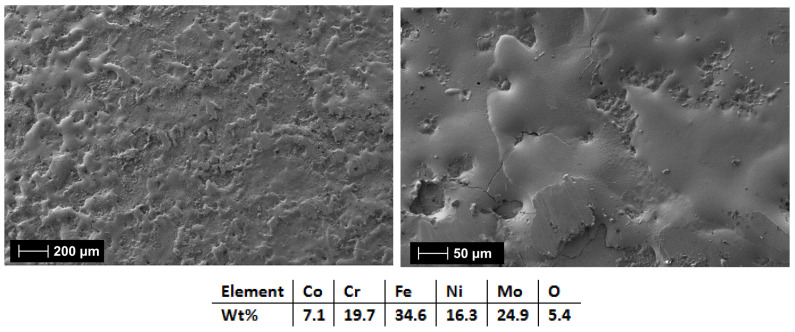
SEM and EDS analysis results for HEA-Co-C1.

The following set of coatings examined were the HEA-Co-C1 (Figure 19) (3 layers) and HEA-Co-C2 (6 layers) produced from the Co_0.5_CrFeNiMo (HEA-Co) electrode. As previously mentioned, it was important to keep a low Co content for this composition due to the critical raw materials situation, but Co alloys present improved properties when exposed to corrosive environments [51].

**Figure 20:**
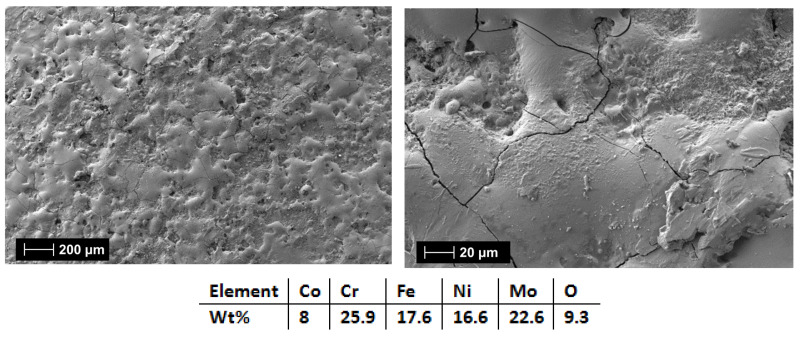
SEM and EDS analysis results for HEA-Co-C2.

For the HEA-Co coatings case, it was observed the recurring effect from the HEA-FREE, where when HEA-Co-C2 (Figure 20) was applied, microfissures were present on the surface along with a higher roughness. HEA-Co-C1 coating (Figure 19) presents a lower count of defects, indicating that the selected parameters and numbers of layers were suitable for this HEA composition. For HEA-Co-C2, besides the microfissures, pores are also present. Such pores are common with the ESD process due to the high temperatures used during the process, where the air bubbles are released during the melting phase. The pores are usually superficial, affecting the last 1–2 layers [59,60]. The EDS analyses indicate a higher oxidation degree for the HEA-Co-C2 coating, similar to the HEA-FREE-C2 case.

**Figure 21:**
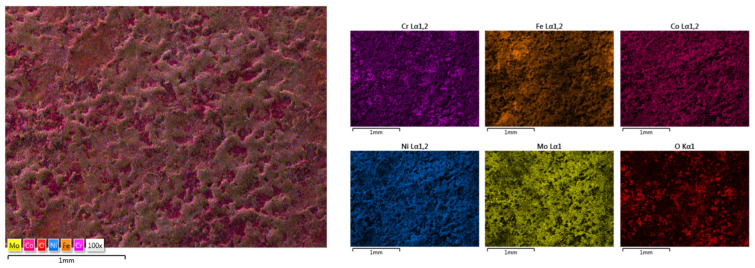
Elemental mapping results for HEA-Co-C1.

**Figure 22:**
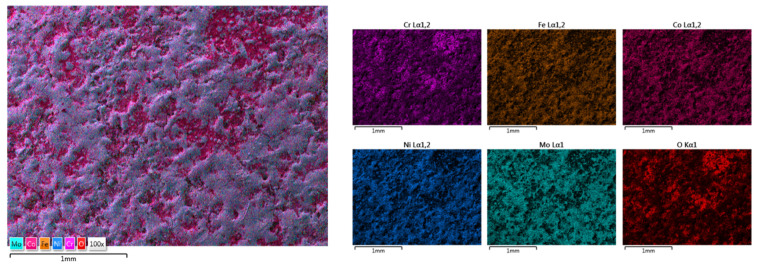
Elemental mapping results for HEA-Co-C2.

The elemental mapping results from Figure 21 for HEA-Co-C1 and Figure 22 for HEA-Co-C2 presented a good elemental distribution across the entire analyzed surface, with O being present for both cases, more abundant where Cr was also identified. Compared to the previous coatings, for this case a higher hardness is to be expected due to the known properties of the Cr_2_O_3_ correlated with a higher wear resistance but also increased corrosion resistance [61].

#### 3.2.3. Al_0.5_CrFeNiMo

**Figure 23:**
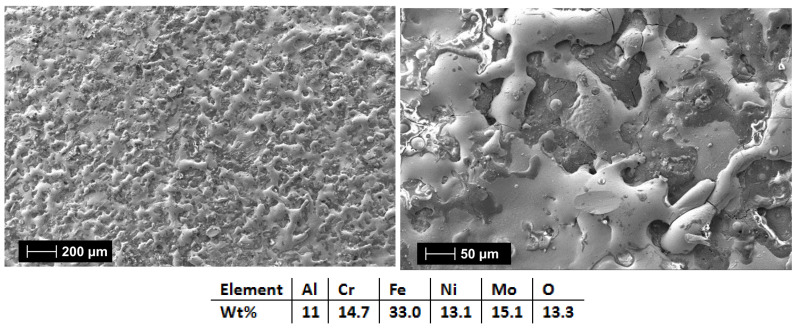
SEM and EDS analysis results for HEA-Al-C1.

**Figure 24:**
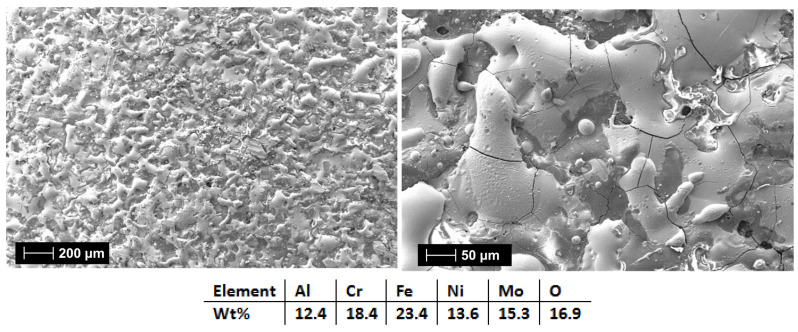
SEM and EDS analysis results for HEA-Al-C2.

The third set of analyzed coatings were for the HEA-Al-C1 (3 layers) and HEA-Al-C2 (6 layers) produced from the Al_0.5_CrFeNiMo (HEA-Al) electrode. Due to the chemical composition of this HEA material, splats are to be expected during the deposition process. Al is known for a significantly lower melting temperature compared to Cr, Fe, Ni, and Mo. The splats do not usually affect the integrity or coating continuity but are to be considered. As observed, in both cases presented in Figure 23 for HEA-Al-C1 and Figure 24 for HEA-Al-C2, rougher surfaces are present. When comparing the 2 coatings, HEA-Al-C1 presents fewer microfissures and fewer open pores. For HEA-Al-C2, a more uniform coating was observed, and empty spaces created by the multiple layers were filled with materials when increasing the number of layers. The EDS analysis results present a higher oxidation for HEA-Al-C2, similar in every set of coatings studied. Generally, as for the other sets, the coating was uniform and continuous.

**Figure 25:**
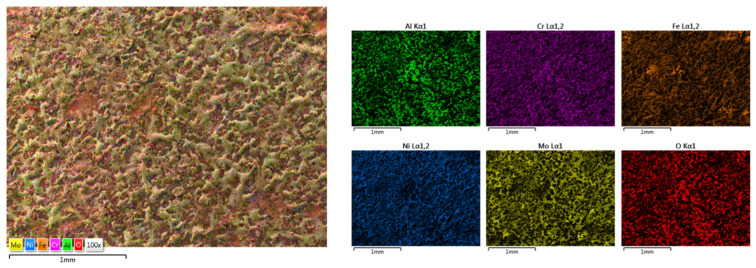
Elemental mapping results for HEA-Al-C1.

**Figure 26:**
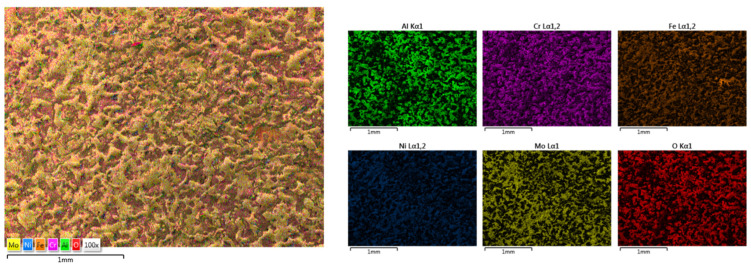
Elemental mapping results for HEA-Al-C2.

The elemental mapping results presented in Figure 25 for HEA-Al-C1 and in Figure 26 for HEA-Al-C2 present a high degree of homogeneity and uniformity globally. It has been noticed that due to the splat formation, the picks appear as separated, where Al is present in abundance in areas where O is also abundant. As for the previous set of samples, a higher wear resistance could be indicated by the presence of Al_2_O_3_, along with corrosion resistance. Fe splats are also observed, with a higher density in smaller areas.

**Figure 27:**
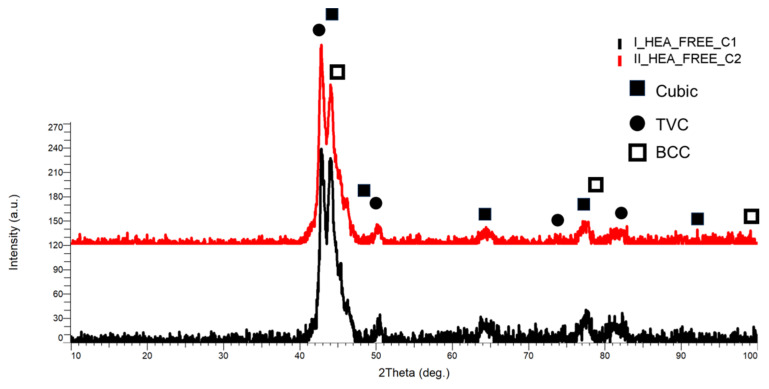
XRD comparative analysis results for the HEA-FREE-C1/HEA-FREE-C2 coated carbon steel samples.

**Figure 28:**
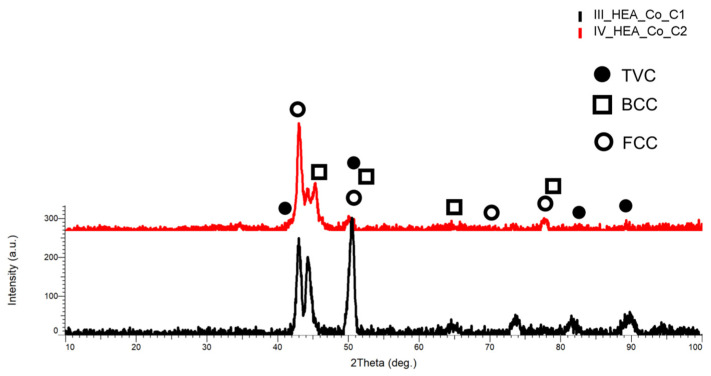
XRD comparative analysis results for the HEA-Co-C1/HEA-Co-C2 coated carbon steel samples.

**Figure 29:**
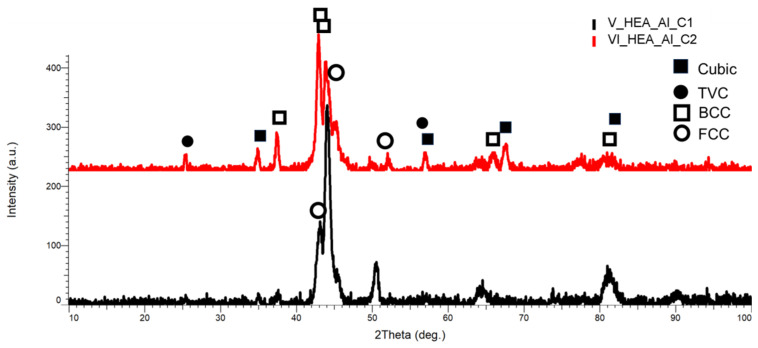
XRD comparative analysis results for the HEA-Al-C1/HEA-Al-C2 coated carbon steel samples.

The XRD analysis results for the HEA coatings are presented in Figure 27 for HEA-FREE-C1/HEA-FREE-C2, in Figure 28 for HEA-Co-C1/HEA-Co-C2, and in Figure 29 for HEA-Al-C1/HEA-Al-C2.

According to the identification performed for the HEA-FREE-C1/HEA-FREE-C2, it was observed that the highest intensity was at the 2θ angular position of 43.2° with a major TVC and cubic phases with an abundance of MoNi and CrMo, together with a secondary peak identified at the 2θ angular position of 45° with a major BCC phase with an abundance of FeMo and CrMo, indicating the presence of the elements and their crystallization after the coating process, confirming the alloying process. Points of interest were also observed at the 2θ angular positions of 50.5°, 64.5°, 77.1°, and 82.13° with an abundance of the principal elements. When comparing the results, it could be observed that the number of layers did not change the crystallization process in an important matter, where the noticeable difference was observed at the secondary peak, which could imply that the secondary peak and phase developed with the layering increase to form a singular peak.

Important differences were observed when analyzing the HEA-Co-C1/HEA-Co-C2 coatings by XRD. When the identification was performed, a switch of maximum intensity was noticed, while for HEA-Co-C1, the highest intensity was at the 2θ angular position of 50.5° with a major FCC, BCC, and TVC phase with an abundance of MoNi, CrFeMo, CrFe, and CrNi, but also CoMoFe, while for HEA-Co-C2 coating, the highest intensity was at the 2θ angular position of 42.11° with a major FCC phase abundant in MoNi. Although the secondary peaks presented a high intensity for HEA-Co-C1, for the principal peak and all the mixture elements were identified also by EDS, the alloying process during all steps was confirmed. From the analyzed coatings, it was observed that the BCC and FCC phases were present as major phases together with a lattice deformation indicated by the TVC phase.

According to the identification performed for the HEA-Al-C1/HEA-Al-C2 coatings, a shift to the left was observed in the highest intensity angular position, while improved crystallization was observed for HEA-Al-C1. With increasing the number of layers, the intensity declined, indicating the possibility of a higher lattice deformation for HEA-Al-C2. According to the VEC calculation, the presence of the BCC phase was noted, being present in the areas with the highest intensity, namely a 2θ angular position of 44.3° for HEA-Al-C1 and 42.02° for HEA-Al-C2, with an abundance of FeMo and AlFe. The FCC phase identified at the 2θ angular position of 43.5° and 50.48° decreased in intensity along with the increase in the number of layers applied to the substrate, suggesting a decrease in ductility according to the theory [55].

This observation is correlated to the microstructures presented in Figure 23, where cracks were present on the coating surface for HEA-Al-C2. The lattice deformation is noted for the HEA-Al-C1/HEA-Al-C2 due to the presence of the cubic and TVC phases, which present an abundance of CrFeMo and CrNi.

It was noted that the number of layers has an important impact on the coating and that for a lower number of layers, a lower oxidation degree was obtained, along with fewer surface defects. Although oxygen was present in all cases, the elemental chemical combination might have a positive effect on the results due to the possibility of obtaining a higher wear and corrosion resistance. Microstructural and crystallinity changes observed in the analysis results play a crucial role in determining the mechanical and physical properties of the studied HEAs. The formation of homogenous phases and reduced defects enhances mechanical strength and wear resistance by limiting the initiation and propagation of microcracks. For the studied case, the microcracks identified on the coating surface are mainly related to the ESD rapid cooling under argon shielding and the variability induced by manual application. The process inherently involves low heat input due to its pulsed nature, where parameter adjusting is required based on the deposition behavior.

## 4. Conclusions

In this paper, the microstructural and chemical aspects of CrFeNiMo, Co_0.5_CrFeNiMo and Al_0.5_CrFeNiMo, were investigated based on the technological processing methods’ results for producing bulk and coated samples by spark plasma sintering and electrospark deposition. This research leads to the following conclusions:

1. The microstructural and chemical analyses revealed an improved elemental distribution, microstructural refinement, and homogeneity correlated to the temperature increment of 50 °C when sintering the HEAs. This result was observed for all trials.

The identified phases for the sintered samples indicated that the crystallographic lattice was severely distorted during the consolidation process, one of the reasons being the high-entropy alloys’ specific effects. The presence of the TVC phase indicated the tetragonal distortion where the absence of the BCC phase for the CrFeNiMo and Co_0.5_CrFeNiMo and the FCC phase for the Al_0.5_CrFeNiMo could be justified.

For the electrodes and coatings production, the candidates for further processing selected were HEA-FREE-S3-1050 (CrFeNiMo HEA sintered at 1050 °C), HEA-Co-S3-1000 (Co_0.5_CrFeNiMo sintered at 1000 °C), and HEA-Al-S3-950 (Al_0.5_CrFeNiMo sintered at 950 °C) due to phase stability and increased hardness.

2. It was observed that the number of layers has an important impact on the coating aspect. When 3 layers were applied, a lower oxidation degree was obtained, along with a lower microfissure count. The layer count had a great impact on the correlation between the intensity of the identified FCC phase and the reduced ductility with the microstructural results where cracks were observed on the sample surface.

3. Further investigations will be conducted on the ESD-coated samples exposed to aggressive media (e.g., simulated geothermal environment) in order to observe the compositional and processing route effect on the corrosion resistance properties.

## Figures and Tables

**Table 1:** SPS parameters used for the HEAs consolidation process.

HEA Composition	Sintered Sample Codification	Pressure (MPa)	Heating Rate (°C/min)	Dwell Time (min)	Cooling Rate (°C/min)	Sintering Temperature (°C)
CrFeNiMo	HEA-FREE-S1-950	50	50	5	30	950
	HEA-FREE-S2-1000	50	50	5	30	1000
	HEA-FREE-S3-1050	50	50	5	30	1050
Co_0.5_CrFeNiMo	HEA-Co-S1-900	50	50	5	30	900
	HEA-Co-S2-950	50	50	5	30	950
	HEA-Co-S3-1000	50	50	5	30	1000
Al_0.5_CrFeNiMo	HEA-Al-S1-850	50	50	5	30	850
	HEA-Al-S2-900	50	50	5	30	900
	HEA-Al-S3-950	50	50	5	30	950

**Table 2:** Electrospark deposition parameters used during experimentation.

Electrode Chemical Composition	Output (μF)	Power (V)	Frequency (Hz)	Ar Atm. (L/min)	Deposition Rate (mm/s)
CrFeNiMo	30	100	360	5	1.86
Co_0.5_CrFeNiMo	20	100	850	5	2.62
Al_0.5_CrFeNiMo	20	100	360	5	2.71

**Table 3:** Density, porosity, and Hardness testing results.

HEA Sample	Avg. Dens. ± Std. Dev. (g/cm^3^)	Theor. Dens. (g/cm^3^)	Densification (%)	Porosity (%)	Avg. Hardness HV2/15 ± Std. Dev.
HEA-FREE-S1-950	8.192 ± 0.007	8.65	94.703	5.297	835.5 ± 21.68
HEA-FREE-S2-1000	8.389 ± 0.006	8.65	96.983	3.017	923.34 ± 2.70
HEA-FREE-S3-1050	8.380 ± 0.003	8.65	96.876	3.124	891.42 ± 8.89
HEA-Co-S1-900	7.624 ± 0.009	8.67	87.933	12.067	493.95 ± 23.17
HEA-Co-S2-950	8.240 ± 0.006	8.67	95.045	4.955	757.7± 5.97
HEA-Co-S3-1000	8.387 ± 0.014	8.67	96.736	3.264	790.74± 6.22
HEA-Al-S1-850	6.195 ± 0.089	7.81	79.325	20.675	192.04 ± 11.11
HEA-Al-S2-900	6.447 ± 0.057	7.81	82.548	17.452	307.68 ± 32.20
HEA-Al-S3-950	6.952 ± 0.010	7.81	89.014	10.986	530 ± 28.73

## Data Availability

The original contributions presented in this study are included in the article. Further inquiries can be directed to the corresponding authors.
